# Clozapine monitoring at a specialised psychiatric hospital: A retrospective chart review

**DOI:** 10.4102/sajpsychiatry.v29i0.2039

**Published:** 2023-10-20

**Authors:** Michelle V. Daniels, Suvira Ramlall

**Affiliations:** 1 Department of Psychiatry, Faculty of Health Sciences, University of KwaZulu Natal, Durban, South Africa

**Keywords:** clozapine monitoring guidelines, clozapine monitoring guidelines in South Africa, treatment-resistant schizophrenia, benign ethnic neutropaenia, benign familial neutropaenia, clozapine-induced neutropaenia, clozapine-induced agranulocytosis

## Abstract

**Background:**

Clozapine is the only Food and Drug Administration (FDA) and National Institute for Care and Excellence (NICE) approved drug for treatment-resistant schizophrenia (TRS). Its potentially life-threatening haematological side effects of neutropaenia and agranulocytosis mandate rigorous monitoring of neutrophil counts, presenting unique, Third-World population challenges.

**Aim:**

To describe the Clozapine white blood cell monitoring practice and outcomes in a local psychiatric hospital.

**Setting:**

At a specialist Psychiatry unit in Durban, KwaZulu-Natal, which follows a modified algorithm of the South African Standard Treatment Guidelines for Clozapine monitoring.

**Methods:**

A retrospective chart review composed of 120 patients on Clozapine treatment from 01 July 2018–31 December 2020. Demographic and clinical information was captured in a Redcap database. Descriptive statistics using categorical variables were used.

**Results:**

The study population was from a low socioeconomic background, with low levels of education and employment. A baseline neutrophil count was recorded in 58 files (48.3%). Clozapine was stopped in 6 out of the 120 patients due to ‘neutropaenia’ (absolute neutrophil counts ranging from 1.18 to 1.6); none developed agranulocytosis. Their duration of Clozapine treatment ranged from 2 weeks–15 years.

**Conclusion:**

Haematological monitoring frequency and documentation of patients receiving Clozapine were not in compliance with the hospital’s adapted guidelines and may have resulted in the termination of treatment before true neutropaenia developed. Patients developed neutropaenia at low doses of Clozapine and after many years of treatment.

**Contribution:**

These results suggest local Clozapine monitoring guidelines should be more strictly adhered to.

## Introduction

Clozapine is the only Food and Drug Administration (FDA)- and National Institute for Care and Excellence (NICE)-approved drug for treatment-resistant schizophrenia (TRS).^1^ However, its well-documented haematological side effects of neutropaenia and agranulocytosis necessitate rigorous blood monitoring for which local^2^ and international^1^ protocols exist.

Schizophrenia is prevalent in approximately 0.3% – 0.7% of the population^3^; the prevalence in South Africa may be higher at 1%.^4^ It is estimated that 30% of patients develop TRS^5^ and this results in higher costs due to longer hospital stays, long-term morbidity and a poorer prognosis.^6,7^

Treatment-resistant schizophrenia is defined as ‘schizophrenia that has not responded adequately to treatment despite the sequential use of at least two antipsychotic drugs’ (at least one of which should be a second‑generation antipsychotic that is not Clozapine).^1^ Clozapine is the gold standard for the treatment of TRS^8^; its use has been shown to lower psychotic relapses significantly and to decrease rehospitalisation rates by 18% – 30%, but these rates should be used with caution as the studies had several limitations in their methodologies.^9^ Clozapine is superior to any other neuroleptic for treating psychosis and yet it is also the most underutilised.^7,10^ This is attributed to Clozapine’s several black box warnings; it can cause the rare but potentially life-threatening haematological side effects of neutropaenia and agranulocytosis.^1^

Neutropaenia is defined as an absolute neutrophil count (ANC) of < 1.5 × 10^9^/L.^11^ It is caused by a decreased production or increased peripheral destruction of neutrophils from a variety of different causes, namely, bone marrow toxicity and autoimmune conditions.^12^ The incidence of Clozapine-induced neutropaenia (CIN) is 1.8%,^12^ which usually manifests within 1–2 weeks post Clozapine. Clozapine is oxidised or activated to an electrophilic, reactive nitrenium ion that binds to neutrophils, causing them to be released from the bone marrow before they have reached maturity. The nitrenium ions are also postulated to cause oxidative stress-induced neutrophil apoptosis, which would decrease the number of circulating neutrophils.^12^ Antineutrophil antibodies may also be generated by nitrenium ions reacting with neutrophil proteins, resulting in hapten formation; hapten, a molecule only capable of causing the production of antibodies when fastened to a larger antigen, has been postulated to be involved in the aetiology of neutropaenia. The degree of neutropaenia depends on the Clozapine dose.^12,13^ The higher the dose, the more likely neutropaenia is to develop.^12,13^

Agranulocytosis is defined as an absence of circulating white cells or leukocytes or an ANC of < 0.5 × 10^9^/L.^12^ The estimated incidence of Clozapine-induced agranulocytosis (CIAG) is < 1%^14^; even though uncommon, it is potentially life-threatening. It usually manifests about 3–4 weeks post-initiation of Clozapine. The mechanism of CIAG is unknown, but recent literature postulates that an autoimmune cause involving the nitrenium ion may be a factor.^12^

Clozapine-induced neutropaenia or CIAG can be prevented by identifying at-risk patients, especially those who are immunocompromised and monitoring them closely. The risk of agranulocytosis rises with age, but the risk of neutropaenia decreases with age.^12^ A low baseline neutrophil count may also be associated with neutropaenia but not agranulocytosis.^12^ Clozapine use in patients with human immunodeficiency virus (HIV), although not recommended, may be useful if their CD4 counts are high.^15^ Whether HIV is a risk factor for agranulocytosis is not known, but it is imperative that HIV-positive patients who require Clozapine be closely monitored and be only managed at a specialist level.^15^ Another risk factor for developing agranulocytosis is the co-prescription of medication that has the propensity to drop blood counts.^15^

Benign familial neutropaenia (BFN), also controversially known as benign ethnic neutropaenia (BEN),^16^ is:

[*T*]he occurrence of a neutropaenia, defined by normative data in white populations, in individuals of other ethnic groups who are otherwise healthy and who do not have repeated or severe infections.^12^ (p. 1)

Benign familial neutropaenia’s pathogenesis may be explained by a genetic polymorphism that affects the expression of the Duffy antigen on erythrocytes.^17^ The incidence is estimated to be up to 25% – 40% of those of African descent outside of the United States.^17^ These individuals have a lower-than-normal ANC and overall white cell count (WCC), yet appear to be at no greater risk of developing agranulocytosis or infection than those without BFN.^17^

These findings support the need for Clozapine monitoring criteria that are sensitive to ethnic differences.^17^ All patients receiving Clozapine are required to be intensively monitored according to one of several existing monitoring guidelines.^17^

According to the NICE and South African Standard Treatment Guidelines (SASTG) and Essential Medicines List for South Africa, after Clozapine initiation, differential WCCs need to be done weekly for 18 weeks to monitor for the development of CIN.^1,2,18^ This is when CIN is most likely to develop. Thereafter, differential counts need to be done every two weeks for a year, and then four weeks thereafter.^1,2,18^ However, the use of modified criteria for CIN and CIAG in BFN patients is of the utmost importance to prevent unnecessary Clozapine discontinuation.^11^ According to the Maudsley Handbook,^13^ Clozapine should be discontinued if the total WCC drops below 4 × 10^9^/L or if the ANC drops below 2.5 × 10^9^/L.^13^ But in BFN patients, the values for discontinuing Clozapine are slightly lower: a leukopenia of < 3 × 10^9^/L and a neutropaenia of < 1.5 × 10^9^/L.^13^ The United Kingdom has adopted these modified criteria, but these criteria have not been adopted in the United States.^17^ The SASTG state that Clozapine should be withdrawn if the ANC drops below 1 × 10^9^/L.^2^ The NICE guidelines are based on a first-world, predominantly white population. To our knowledge, it has not been determined if the same risk of developing neutropaenia or agranulocytosis exists in our ethnically diverse South African population.

The authors have anecdotally observed that weekly blood tests have significant cost and compliance implications for both hospitals and patients. The drawing of blood is painful and unpleasant for patients. Travel costs to the hospital are prohibitive for outpatients, especially for mental healthcare users who are often social grant recipients. For hospitals, each patient visit has cost implications for human resources, time and consumables. These factors are likely to negatively impact adherence to rigorous monitoring protocols.

The cost-effectiveness of various methods of monitoring white blood cells was assessed in the United Kingdom, the United States and two European countries, and these methods were compared to no-monitoring.^18^ The results showed that all four methods of monitoring increased quality-adjusted life years by less than 1 day per patient, compared to no-monitoring of white blood cells. This meant that more than 5000 patients would need to be monitored to avoid one death.^18^ They recommended that public health authorities revise their policies as WCC monitoring in patients who were on Clozapine was not found to be cost-effective.^18^

Treatment-resistant schizophrenia occurs in about one-third of patients^5^; therefore, approximately one-third of patients with schizophrenia should be on Clozapine. The prevalence of BFN in the African population is unknown; the threshold for classifying patients with neutropaenia or agranulocytosis may be lower than that which is recommended in international guidelines. As the literature has not found monitoring of white blood cell count to be cost-effective in populations outside of Africa, it is important from a clinical and financial perspective to establish the state of South African haematological monitoring practices. This study therefore aimed to describe the Clozapine white cell monitoring practice in a local psychiatric hospital.

## Research methods and study design

### Study design and setting

This study was a descriptive, retrospective chart review and was conducted at a specialised psychiatry unit in Durban, KZN.

### Study population and sampling strategy

The files of adult patients, over 18 years old, who were receiving Clozapine during the study period were included in the analysis. Inclusion criteria were files of all patients who were on Clozapine regardless of the indication. All files were included until the required sample size was reached. The study site has its own Clozapine monitoring guidelines (King Dinuzulu Clozapine Clinic Standard Operating Procedures, personal communication) which is a modified algorithm of the SASTG: Clozapine would be stopped if a patient’s ANC is < 1 × 10^9^/L, but only if this drop occurs after 18 weeks of Clozapine treatment (King Dinuzulu Clozapine Clinic Standard Operating Procedures, personal communication). The hospital guidelines (King Dinuzulu Clozapine Clinic Standard Operating Procedures, personal communication) also state that trimonthly tests, as opposed to monthly blood tests, may be done with signed informed consent if the patient is medically and psychiatrically stable.

### Data analysis

Descriptive statistics were used to summarise the data. Frequencies and percentages were used for categorical data. Frequency distributions of numeric data were examined for normality, and mean standard deviation (s.d.) or medians of the interquartile range (IQR) were used as appropriate.

Compliance with guidelines was determined by meeting a set of criteria as predetermined by the aforementioned NICE and SASTG Clozapine monitoring guidelines.^1,2,18^ Categorical variables associated with compliance were identified using a chi-squared test. Variables measured on a numeric scale were compared using a *t*-test. A sample size of 114 patients was necessary to estimate the proportion of patient monitoring which is compliant with treatment guidelines to within 13% with a probability of 95% and assuming baseline compliance of 50%. The *p*-value was set to 0.05. The sample size was calculated, and the data were analysed using Stata V13.1 statistical software.

### Ethical considerations

Ethical approval was obtained from the Biomedical Research Ethics Committee of the the University of KwaZulu-Natal (BREC/00002262/2020) and the KwaZulu-Natal Department of Health. Permission was granted by the Ethics Committee of the study site to conduct the research.

## Results

A total of 120 clinical records were reviewed. The majority of the patient samples were black African people, mixed race people and Indian or Asian people (87.5%), predominantly male (*N* = 83; 69.2%), with the mean age of the total sample being 43.97 years (s.d. ± 14.22) and a median of 43.0 years. The demographic profile of subjects is summarised in Table 1. The highest level of education (HLOE) was high school (*N* = 81; 77.9%), with the majority (*N* = 72; 88.9%) having not completed matric (Grade 12) and nine (7.5%) having completed tertiary education; there was also a high unemployment rate of 98%; and 95% of the sample were single.

**TABLE 1:** Demographic profile of the study population (*N* = 120).

Demographics	*n*	%
Male	83	69.2
Female	37	30.8
**Race**		
Black African people	27	22.5
Mixed race people	12	10.0
Indian or Asian people	66	55.0
White people	15	12.5
**Home language**		
English	93	77.5
isiZulu	27	22.5
**Highest level of education**		
Primary school	14	13.5
High school	81	77.9
Tertiary	9	8.7
Unknown		
**Employment status**		
Employed	3	2.5
Unemployed	98	83.1
Pensioner	17	14.4
Unknown	2	1.7
**Marital status**		
Single	95	79.8
Co-habiting	4	3.4
Divorced	6	5.0
Widow or widower	10	8.4
Unknown	1	0.8
Smoker	57	47.5
Non-smoker	63	52.5

Table 2 summarises the recorded haematological monitoring data. Complete monitoring data were not available for all 120 participants. Of the 120 files reviewed, half (*N* = 60) recorded a baseline total WCC, and 48.3% of the files recorded a baseline neutrophil count. Haematological data at the last recorded visit during the study period were available in 93.3% (WCC) and 95% (neutrophil count) of clinical records. Twenty-four files (20%) had recorded the initial neutrophil count; 2 files (1.7%) had the biweekly neutrophil counts monitored for 12 months; and 27 files (22.5%) had monthly neutrophil counts documented 12 months after Clozapine was initiated. None of the 120 patients in the sample had evidence of BFN, i.e. no patients had a low baseline neutrophil count.

**TABLE 2:** Haematological monitoring of Clozapine.

Haematological monitoring parameters	Recorded in file (*N* = 120) (%)		Mean	Standard deviation	Median	Range
	*n*	%				
Baseline WCC	60	50.0	8.05	2.52	7.91	3.19–16.7
WCC count at last recorded visit	112	93.3	7.76	2.66	7.28	3.36–16.7
Baseline neutrophil count	58	48.3	5.1	1.89	5.08	2.36–11.8
ANC at last recorded visit	114	95.0	4.76	1.8	4.61	1.25–11.2
ANC recorded weekly for 18 weeks	24	20.0	-	-	-	-
ANC recorded weekly for duration of study	46	38.3	2.85	3.72	0	0–13
ANC recorded 2 weekly for 12 months	2	-	-	-	-	-
For those that were recorded incompletely	15	-	0.35	1.23	0	0–10
ANC recorded monthly after 6 months of treatment	27	-	-	-	-	-
For those that were recorded incompletely	80	-	4.71	2.61	4	0–10

ANC, absolute neutrophil count; WCC, white cell count.

Table 3 provides definitions of neutropaenia as per the different guidelines referenced in this article.

**TABLE 3:** Neutropaenia definitions as per guidelines referenced.

Guidelines	Definition
NICE	ANC < 2.5 × 10^9^/L For BFN patients, ANC < 1.5 × 10^9^/L.
SASTG	ANC < 1 × 10^9^/L
KDHC†	ANC < 1 × 10^9^/L but only if the drop occurs after 18 weeks of Clozapine treatment

† , King Dinuzulu Clozapine Clinic Standard Operating Procedures, personal communication.

NICE, National Institute for Care and Excellence; ANC, absolute neutrophil count; BFN, benign familial neutropaenia; SASTG, South African Standard Treatment Guidelines; KDHC, King Dinuzulu Hospital Complex.

Table 4 provides details of the six (0.05%) patients who developed ‘neutropaenia’ (i.e. whose Clozapine was stopped due to concerns about decreasing neutrophil counts or whose reason for stopping Clozapine was recorded as neutropaenia by the treating clinician). All six participants were black African people (*N* = 4) and Indian people or Asian people (*N* = 2). All baseline blood results were within normal ranges. Clozapine doses ranged from 50 mg to 475 mg total daily doses. Patients 1–3 were based on the NICE guidelines (ANC < 1.5); patients 4, 5 and 6 had Clozapine discontinued at ANCs of 1.5, 1.6 and 1.18, respectively. With patient 5, Clozapine was not discontinued; rather the differential count was repeated, and when the neutrophil count was > 3.0 in the following month, the result was deemed a laboratory error. Clinical records indicated ‘neutropaenia’ as a reason for discontinuing Clozapine in patient 6. Patients 1–4 developed neutropaenia between 5 and 15 weeks of initiation of Clozapine. If the hospital’s guidelines were used, none of these patients would have had their Clozapine stopped as none of their ANCs dropped below 1 × 10^9^/L. The ANC was not recorded in three of these patients’ files after Clozapine was stopped to check if the differential count had returned to normal. The two that were, were done a week after stopping Clozapine and the ANCs had returned to within the normal ranges. Patients 1 and 2 were also administered sodium valproate, which is also haematotoxic. Based on the six patients, one in three (33.3%) patients were likely to develop neutropaenia if administering both Clozapine and sodium valproate, and this was statistically significant (*p* = 0.03, using a binomial probability test).

**TABLE 4:** Patients who had Clozapine discontinued due to ‘neutropaenia’.

Neutropaenic patient details	Patient 1	Patient 2	Patient 3	Patient 4	Patient 5	Patient 6
**Demographics**						
Age	34	45	43	26	45	60
Sex	M	M	F	M	M	F
Ethnicity	Black African person	Black African person	Black African person	Black African person	Indian or Asian person	Indian or Asian person
Diagnosis	SAD: BST	TRS	SAD: BST	TRS	TRS	TRS
**Baseline**						
Date	27 August 2019	19 February 2020	05 April 2019	14 February 2020	17 February 2015	18 February 2004
WCC	5.15	7.06	3.46	4.76	8.2	5.9
ANC	2.5	5.46	5.21	2.77	4.21	3.3
**Blood results when neutropaenia developed**						
Date	18 December 2019	25 March 2020	25 June 2019	03 April 2020	N/A – Clozapine not stopped	17 September 2019
WCC	4.5	3.13	4.2	3.36	8.5	3.86
ANC	1.39	1.5	1.25	1.47	1.6	1.18
Dose neutropaenia developed	175/300	50/100	100/100	50 nocte	100/200	100/175
**Blood test after stopping Clozapine**						
Date	24 December 2019	Not recorded	Not recorded	Not recorded	N/A but blood tests repeated following month	24 September 2019
WCC	4.61	Not recorded	Not recorded	Not recorded	3.66	5.43
ANC	2.31	Not recorded	Not recorded	Not recorded	2.4	2.86
Other meds	Zuclopenthixol depot 400 mg IMI monthly Epilim 500/800	Zuclopenthixol depot 200 mg IMI monthly Epilim 500/500	Citalopram 20 daily	Nil	Nil	Nil
Other side effects noted	Weight gain (16 kg in 4/12) Constipation Sialorrhoea Dyslipidaemia	Sedation Weight gain Dyslipidaemia	Constipation Sedation Weight gain Dyslipidaemia	Constipation Tachycardia	Dyslipidaemia Weight gain	Nil
**Duration of CLZ treatment at time of neutropaenia** †	4 months	Within 1 month	Within 2 months	Within 2 weeks	5 years	15 years later

M, male; F, female; SAD:BST, seasonal affective disorder: Bright light therapy; TRS, treatment-resistant schizophrenia; ANC, absolute neutrophil count; N/A, not applicable; WCC, white cell count; CLZ, Clozapine.

† , Neutropaenia develops usually within first 2 weeks of Clozapine initiation, so the times most of the patients developed neutropaenia was unusual.

The black African, mixed race and Indian and/or Asian patients had a lower mean baseline WCC than the white participants. The black African patients were noted to have had a statistically significant lower mean ANC of 3.59, s.d. ± 1.73 (*P* < 0.05).

Repeat differential counts were not recorded for three of the six patients to determine if their neutrophil counts had changed after Clozapine had been stopped. For the other three patients, two had their blood tests repeated within 1 week, and one had their blood tests repeated within 1 month, at which time all three of their ANCs had normalised.

## Discussion

Clozapine is the only evidence-based and approved drug for the treatment of TRS. However, it has a rigorous haematological monitoring protocol due to its risk of neutropaenia and agranulocytosis. The Clozapine monitoring practice in a public specialist psychiatric hospital was therefore explored to determine the adherence to recommended guidelines and the frequency of haematological effects.

The study consisted of participants, mainly of Indian people or Asian people (55%) and black African people (22.5%). Most international studies were conducted in predominantly white populations.^1,4,5,13^ This precluded a direct comparison of our results with international literature. It is well recognised that multiple ethnic and racial factors significantly affect the interindividual variability of the pharmacodynamics and pharmacokinetics of drugs.^19^ This study also highlights the need for pharmacokinetic and pharmacodynamic data from black African, mixed race, Indian/Asian and other patient populations. This need has been recognised by the FDA, which published the Enhancing the Diversity of Clinical Trial Populations – Eligibility Criteria, Enrolment Practices, and Trial Designs guidance document in November 2020.^20^ This document aims to promote the widening of eligibility criteria in study designs to allow for participants to better reflect the population being studied by increasing the enrolment of underrepresented populations.^20^

The study site utilised an adapted version of the SASTG for Clozapine monitoring; however, less than a quarter of our sample was being haematologically monitored according to these or the NICE guidelines. Comparative figures for other local or international clinical sites were not available. While the reasons for the low monitoring rate could not be ascertained from a retrospective file review, possible reasons could be missing or unrecorded information, clinicians not having enough time to do blood tests on these patients, and even patients defaulting on their clinic follow-up appointments. Another contributory factor was the Severe acute respiratory syndrome coronavirus 2 (SARS-CoV-2) or the COVID-19 pandemic, which decreased patients’ clinic visits in general.

Rigorous monitoring has been reported to not be cost-effective and public health authorities in the United Kingdom, the United States and several European countries were recommended to revise their Clozapine monitoring protocols.^18^ Given the severe local financial constraints, it is relevant to establish whether the potential risk to and magnitude thereof to patients on Clozapine justify the costs associated with regular haematological monitoring. Using data from a simulated cohort of 100 000 patients, Giradin et al. concluded that the total annual cost per patient of a no-monitoring strategy for patients on Clozapine was $13 173.00, while the total annual costs using the United States’ monitoring system was $14 156.00.^18^ As expected, the costs of monitoring patients on Clozapine increased the more frequent the monitoring occurred, and because the prevalence of sepsis due to agranulocytosis was so low, these costs were not offset by a decrease in costs associated with treating infections.^18^

In the sample, 2.5% of the participants developed CIN. While this falls within the normal range defined for South Africa, the small sample size and the shortcomings identified in the monitoring practices at the hospital must be considered.^19^ The incidence of CIAG both internationally and locally is 0.8%,^1^ and none of the participants in the sample developed CIAG. Geldenhys et al.^19^ in a retrospective cohort study in the Western Cape, South Africa, in 2021, reported an incidence of 0.38% for CIN and 0.19% for CIAG, lower than found in previous studies. However, the authors did not compare the ethnicities of their participants as they did not want the possibility of BFN to be a confounder.^19^ No known similar study in a racially diverse population has been done locally or internationally. These results suggest that the local incidence of CIN and CIAG may be lower than internationally. More studies are necessary to confirm this as it may indicate a need to review local Clozapine monitoring practices to be less stringent, as is practised in the United Kingdom.^7,13^

In this study, BFN was not a factor as baseline white cell and neutrophil counts in the entire sample were within normal ranges. Local guidelines stipulate a lower threshold of discontinuing Clozapine than international standards: an ANC of < 1 × 10^9^/L^2^ compared to the NICE guidelines of < 2.5 × 10^9^/L, respectively.^13^ This suggests that local guidelines have taken BFN into account (i.e. to only discontinue Clozapine if agranulocytosis, rather than neutropaenia, develops). If local guidelines (either SASTG or the study site’s guidelines) were used in the study, the incidence of neutropaenia would have been 0%, and all patients in the sample would have remained on Clozapine. The Maudsley guidelines do offer haematological guidelines for monitoring what they describe as ‘benign ethnic neutropaenia patients’ on Clozapine – but this is a recommendation, not an official standard operating procedure (the cutoff for neutropaenia is lowered to a neutrophil count of < 1.5 × 10^9^/L).^13^ The FDA’s published guidelines are not accessible outside of the United States.^20^

Six participants (0.05%) in this study developed neutropaenia as defined by the Maudsley Handbook and NICE guidelines, which state that Clozapine should be discontinued if the ANC drops below 2.5 × 10^9^/L.^13^ It is not known if the clinicians treating the six patients who developed neutropaenia were guided by the Maudsley Handbook or not, but if the SASTG were adhered to, none of these patients would have needed to have their Clozapine discontinued.

As recorded in Table 4, patients 1–4 developed ‘neutropaenia’ between 5 and 15 weeks of Clozapine initiation, and patients 5 and 6 developed ‘neutropaenia’ after several years. Clozapine-induced neutropaenia usually manifests 1–2 weeks post-initiation of Clozapine^11^; the degree of neutropaenia depends on the Clozapine dose and CIAG usually manifests about 3–4 weeks post-initiation of Clozapine.^7^ The drop in ANCs was not dose-related as these ANCs dropped between daily Clozapine doses of 50 mg and 475 mg, and CIN is associated with higher Clozapine doses.^13^ Notably, the two patients who developed late-onset ‘neutropaenia’ were Indian people or Asian people. The possible role of genetic factors would require exploration in larger sample sizes. Patient 5’s result may have possibly been a laboratory error as there was just an isolated low neutrophil count.

At a specialised hospital, where this study was conducted, patients often have severe forms of mental illness, with medical and substance disorders as co-morbidities; polypharmacy is therefore not uncommon. The possible role of other psychotropic agents impacting the haematological profile of patients must also be considered in patients receiving Clozapine. Our Findings indicated that two of the six patients were also administered sodium valproate; this confounds the attribution of the haematological abnormalities to Clozapine as this anticonvulsant and/or mood stabiliser is known to have adverse haematological effects as well.^10^

### Limitations and recommendations

The study is limited by the small sample size and the quality of the available records. The findings may not be generalisable to other hospitals in the country or to samples with different demographic profiles.

Consensus needs to be reached on which monitoring guidelines are appropriate for the local context and what normative values are appropriate and sensitive to different race groups. The evidence suggests that haematological monitoring is not cost-effective, but in the best interest of patient care, a revised monitoring protocol should be considered to prevent any fatal occurrences of BFN. Local guidelines regarding haematological monitoring of patients on Clozapine should also be reviewed to include recognition of the phenomenon of BFN. This is evidence-based practice in the United Kingdom for patients at risk of developing BEN, who have a lower white cell and the absolute neutrophil cutoff for withdrawing Clozapine. The South African guidelines should also be revised to guide decision-making in the context of co-prescribed drugs that also have haematological side effects. Haematological monitoring guidelines should also be standardised, and one guideline should be adopted by the National Department of Health, so all patients receive the same standard of care throughout the country.

## Conclusion

The haematological monitoring frequency and documentation of patients receiving Clozapine were not in compliance with the hospital’s adapted guidelines and may have resulted in premature termination of treatment. Contrary to the literature, patients developed neutropaenia at low doses of Clozapine and after many years of treatment. The choice of treatment guidelines influences the decision to stop Clozapine treatment.
